# Effectiveness of the bone ring technique and simultaneous implant placement for vertical ridge augmentation: a systematic review

**DOI:** 10.1186/s40729-020-00280-0

**Published:** 2020-12-12

**Authors:** Luis Miguel Sáez-Alcaide, Jorge Cortés-Bretón Brinkmann, Luis Sánchez-Labrador, Fabián Pérez-González, Pedro Molinero-Mourelle, Juan López-Quiles

**Affiliations:** 1 Department of Dental Clinical Specialties, Faculty of Dentistry, Complutense University of Madrid, Plaza Ramón y Cajal S/N, 28040 Madrid, Spain; 2 Department of Conservative Dentistry and Orofacial Prosthodontics, Faculty of Dentistry, Complutense University of Madrid, Madrid, Spain; 3 Department of Reconstructive Dentistry and Gerodontology, School of Dental Medicine, University of Bern, Bern, Switzerland

**Keywords:** Vertical ridge augmentation, Alveolar ridge defects, Bone ring technique, Simultaneous implant placement

## Abstract

**Background:**

Dimensional changes after dental extraction frequently lead to situations in which bone augmentation procedures are required prior to dental implant placement. Bone ring technique (BRT) has been described as a one-stage approach to restore vertical alveolar ridge defects, in which an autogenous or allogeneic cortico-cancellous bone block graft is stabilized with a dental implant inserted simultaneously. The objective of this systematic review was to evaluate the clinical performance of BRT.

**Materials and methods:**

This review was conducted according to PRISMA guidelines. An electronic search was conducted in four databases: (1) The National Library of Medicine (MEDLINE/PubMed) via Ovid; (2) Web of Science (WOS); (3) SCOPUS; and (4) Cochrane Central Register of Controlled Trials (CENTRAL). The Newcastle-Ottawa Quality Assessment Scale and The Joanna Briggs Institute Critical Appraisal tool were used to assess the quality of evidence in the studies reviewed.

**Results:**

Sixteen studies with a total of 186 patients treated with 219 bone rings bocks were included in the review. The studies showed a mean bone gain of 4.94 mm, mean bone resorption of 0.83 mm, and mean marginal bone loss of 0.57 mm after a mean follow-up period of 13.35 months. A mean bone ring survival rate of 97.26% and implant survival rate of 94.97% were recorded.

**Conclusions:**

BRT would appear to be an adequate alternative technique for restoring single vertical alveolar ridge defects with simultaneous dental implant placement. However, further studies comparing this technique with other vertical ridge augmentation procedures in different clinical scenarios are needed to confirm the present results.

## Introduction

Numerous studies have reported dimensional changes in the alveolar bone after dental extraction [1, 2]. It is well known that the alveolar ridge is rapidly reabsorbed during the first 6 months after dental extraction and several factors such as the presence of periodontal disease, periapical pathology, trauma, or the patient’s systemic condition can increase resorption even before tooth loss [3]. These changes lead to alveolar bone defects making the long-term function and esthetic success of rehabilitations with dental implants a challenge [4].

Commonly, an atrophic or severely deficient edentulous ridge will require bone augmentation either simultaneous to implant placement or in a staged approach [5]. Surgical procedures for horizontal bone augmentation have been studied with high predictable results, low complication rates, and implant survival rates of 97–100% [5, 6]. However, vertical ridge augmentation is a more biologically demanding technique and has been associated with higher complications rates and less predictable results due to its high sensitivity [7]. In addition, these augmentation procedures often require a staged approach, as in alveolar osteogenesis distraction, guided bone regeneration (GBR), or reconstruction with bone blocks, which involve high morbidity and longer treatment time [7, 8].

In order to overcome these drawbacks, the bone ring technique (BRT) has been described as a one-stage approach for vertical ridge augmentation, in which an autogenous or allogeneic cortico-cancellous bone block graft is stabilized with a simultaneously inserted dental implant [9]. Several case reports and case series using BRT have been published but there is not enough evidence for its long-term efficacy [10–12].

To the best of our knowledge, no previous reviews have evaluated the clinical outcomes of BRT. Therefore, the aim of this systematic review was to assess the clinical performance of BRT, in terms of bone block and dental implant survival rates, bone gain, bone resorption, marginal bone loss, and complications.

## Materials and methods

### Review development and PICO question

The systematic review was designed in accordance with the PRISMA (Preferred Reporting Items for Systematic Review and Meta-Analyses) statement [13] with the following PICO (Population, Intervention, Comparison, Outcome) model:

- *Population:* systemically healthy edentulous and partially edentulous patients.
- *Intervention:* vertical ridge augmentation with BRT and simultaneous implant placement.
- *Comparison:* other vertical alveolar ridge augmentation procedures at dental implant sites.
- *Outcome:* clinical performance of BRT in terms of bone block and dental implant survival rates, bone gain, bone resorption, marginal bone loss, and complications.

The PICO question was

“In situations in which vertical ridge augmentation is required to restore partially or fully edentulous patients (population), what is the clinical performance (outcome) of bone ring technique (intervention) compared with other vertical augmentation procedures (comparison)?”

### Eligibility criteria

The inclusion criteria were (1) human clinical studies including randomized controlled trials, prospective studies, retrospective studies, case series, and case reports; (2) studies in which interventions aimed to restore fully or partially edentulous patients using BRT with simultaneous implant placement; (3) any publication date; and (4) studies written in English, German, or Spanish (4).

The following outcomes were evaluated: (1) bone gain and bone resorption after BRT; (2) survival rates of bone ring blocks and implants placed after BRT; (3) marginal bone loss around implants placed with BRT; (4) associated complications.

Exclusion criteria were (1) animal studies, in vitro studies, finite element studies, review articles, technical notes and; (2) studies for which the full text was not available.

### Search strategy

An electronic search was conducted for studies published up to 16th August 2020 in four databases: (1) The National Library of Medicine (MEDLINE/PubMed) via Ovid; (2) Web of Science (WOS); (3) SCOPUS; and (4) Cochrane Central Register of Controlled Trials (CENTRAL). Two independent researchers (LMSA, LSL) made the search. The search strategy (adapted to each database) was as follows: ((“bone and bones” [MeSH Terms] OR (“bone” [All Fields] AND “bones” [All Fields]) OR “bone and bones” [All Fields] OR “bone” [All Fields]) AND ring [All Fields]) AND (“dental implants” [MeSH Terms] OR (“dental” [All Fields] AND “implants” [All Fields]) OR “dental implants” [All Fields] OR (“dental” [All Fields] AND “implant” [All Fields]) OR “dental implant” [All Fields]). The electronic search was complemented by a manual search in Oral & Maxillofacial Surgery and Implant Dentistry related journals and in the reference sections of the studies reviewed. To perform the screening process all the references were included into EndNote X9 Library (Clarivate Analytics, Philadelphia, PE, USA).

### Data collection

After the electronic database search, the studies and references identified were screened independently by two pre-calibrated independent reviewers (FPG and PMM). After duplicates and triplicates were removed, the titles and abstracts of the remaining articles were checked for relevance. Any disagreement in the selection process was resolved by discussion with a third reviewer (JCBB). Data from each included article was collected by the reviewers (LMSA and LSL) working together and entered on an Excel spreadsheet (Version 15.17, Microsoft Inc. 2015), including the following: authors, year of publication, study design, number of patients, number of implants, intervention, follow-up, outcomes evaluated, and complications.

### Risk of bias in individual studies

The methodological quality of the included studies was assessed by two independent reviewers (LMSA, LSL). Any disagreement was solved by a third reviewer (JCBB).

Newcastle-Ottawa Quality Assessment Scale tool [14] was used to assess the quality of observational studies, which included a questionnaire divided into 3 categories: selection (4 questions), comparability (1 question), and exposure (3 questions). Each study could obtain a maximum of nine stars. The studies were classified in good, fair or poor-quality (GQ, FQ, or PQ) following the score algorithm proposed by the Agency for Healthcare Research and Quality [15].

The Joanna Briggs Institute Critical Appraisal tool for case reports [16] was used to assess risk of bias in case reports. This includes eight questions; a low risk of bias was considered when ≥ 50% of the answers were “yes,” high risk when ≥ 50% were “no”, and uncertain risk of bias if ≥ 50% of the answers were “unclear.”

## Results

### Study selection (Fig. 1)

**Fig. 1:**
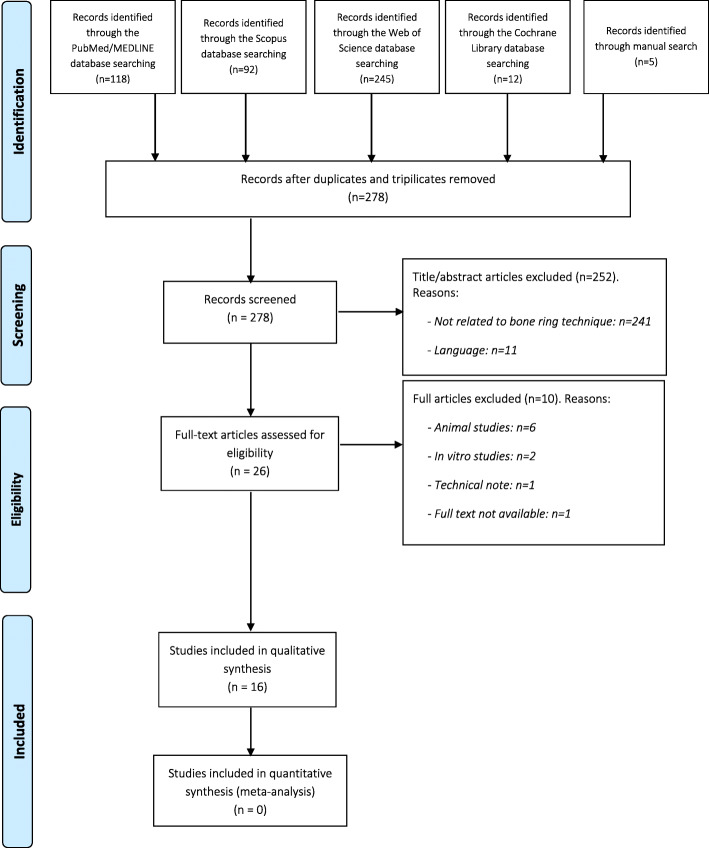
PRISMA flow diagram of the screening and selection process

The initial search yielded 472 references, resulting 278 when duplicates and triplicates were discarded. After title and abstract screening, 252 articles were excluded: 241 studies because they were not related to BRT, while the other 11 were not written in English, Spanish or German. After reading the full text of the 26 selected articles, 10 were discarded for the following reasons: they were animal studies (*n* = 6); in vitro studies (*n* = 2); technical note (*n* = 1); or the full text was unavailable (*n* = 1). Finally, 16 articles were included in the review. The PRISMA Flow diagram in Fig. 1 illustrates the study selection process. Information about the studies reviewed is summarized in Tables 1 and 2. The studies included were non-randomized comparative clinical trials (*n* = 2); prospective studies (*n* = 3); retrospective studies (*n* = 2); case series (*n* = 4); and case reports (*n* = 5). All studies were published between 2005 and 2020.

**Table 1:** Study characteristics

Author-year	Type of study	***N***	***N***, IOI	Intervention	Follow-up
Chandra et al. 2019 [10]	Non randomized comparative clinical study	34	34	Autogenous bone ring (chin) (BR group) vs sticky bone + collagen membrane (SB group) IOI at 6 months	6 months
Flanagan et al. 2016 [17]	Case series	8	8	Allogenic bone ring + IOI + allograft + collagen membrane	1 year
Fukuda et al. 2005 [18]	Case series	9	13	Autogenous bone ring (chin) + IOI	3.1 years
Giesenhagen et al. 2018 [19]	Case report	1	1	Allogenic bone ring + IOI + collagen membrane	6 months
Giesenhagen et al. 2019 [20]	Case series	3	7	Allogenic bone ring + IOI + collagen membrane	36 months
Giraddi et al. 2017 [21]	Prospective study	14	15	Autogenous bone ring (chin) + IOI + PRF membrane	9 months
Miller J. 2019 [22]	Case report	1	1	Allogenic bone ring + xenograft + IOI + collagen membrane	6 months
Nord et al. 2019 [23]	Retrospective study	51	81	Allogenic bone ring + autogenous bone chips + IOI + collagen membrane	12 months
Omara et al. 2016 [24]	Prospective study	10	12	Autogenous bone ring (chin) + IOI	6 months
Peñarrocha et al. 2005 [25]	Case report	1	3	Autogenous bone ring (ramus) + IOI	2 years
Rizzo et al. 2017 [26]	Case series	4	4	Crestal sinus floor elevation with autogenous bone ring (chin) and simultaneous IOI	3 years
Sindel et al. 2018 [27]	Retrospective study	10	10	Sinus floor elevation with autogenous bone ring (chin) and simultaneous IOI	24.3 months
Stevenes et al. 2010 [28]	Case report	1	4	Autogenous bone ring (chin) + IOI	6 months
Tekin et al. 2011 [29]	Case report	1	1	Autogenous bone ring (chin) + IOI	1 year
Wychowansky et al. 2020 [11]	Non randomized comparative clinical study	30	60	Autogenous bone ring (chin) + simultaneous IOI (BR group) vs xenograft tunnel (XG group) and delayed IOI after 6m	24 months
Yuce et al. 2019 [12]	Prospective study	8	12	Autogenous bone ring (chin) + IOI	18 months

*N* number, *IOI* implant, *PRF* platelet-rich fibrin

**Table 2:** Results of individual studies

Author, year	Bone density	Bone gain	Bone resorption	MBL	Survival rate	Other findings	Complications
Chandra et al. 2019 [10]	*a) BR group:* 596.2 ± 115.2 HU *b) SB group:* 659.6 ± 133.8 HU *(p < 0.001)	*a) BR group:* 3.09 mm (buccal) and 3.31 mm (lingual) *b) SB group:* 1.90 mm (buccal) and 1.99 mm (lingual) *(p < 0.001)	–	–	*a) BR group:* 88,23% *b) SB group:* 100%	Implant Stability Quotient (ISQ) *a) BR group:* 61.60 ± 8.9 *b) SB group:* 45.02± 6.33 *(p < 0.034) Histological analysis *a) BR group:* 50.39% ± 11.96% *b) SB group:* 38.91% ± 12.22% *(p < 0.001)	*a) BR group:* -2 IOI and BR failure -1 dehiscence -3 swelling *b) SB group:* -1 dehiscence -3 swelling
Flanagan et al. 2016 [17]	–	–	–	–	100%	–	No complications
Fukuda et al. 2005 [18]	–	–	–	–	92.3%	Radiological stability between baseline and 1.3 years (periapical Rx)	1 IOI and BR failure
Giesenhagen et al. 2018 [19]	–	–	–	–	100%	Radiological stability between baseline and 6 m (panoramic Rx)	No complications
Giesenhagen et al. 2019 [20]	–	–	–	–	100%	Clinical and radiological stability between baseline and 2 year (panoramic Rx)	–
Giraddi et al. 2017 [21]	–	-Mesial bone gain: 3.70 ± 1.10 mm -Distal bone gain: 3.69 ± 1.10 mm	-Mesial bone resorption: 0.73 ± 0.38 mm -Distal bone resorption: 0.78 ± 0.23 mm	–	93.33%	–	1 IOI and BR failure
Miller J. 2019 [22]	–	–	–	–	100%	Radiological stability between baseline and 6 m (periapical Rx) ISQ 68 at 6 m	No complications
Nord et al. 2019 [23]	–	–	–	0.43 mm	97.5%	–	2 IOI failure
Omara et al. 2016 [24]	-Mesial aspect: 420.43 HU -Distal aspect: 325.28 HU	13.07 ± 1.37 mm	0.26 ±0.86 mm	–	100%	–	2 transient numbness of lower lip
Peñarrocha et al. 2005 [25]	–	–	–	–	100%	Radiological stability between baseline and 2 years (panoramic Rx)	No complications
Rizzo et al. 2017 [26]	–	–	–	z	100%	Radiological stability between baseline and 6 years (periapical Rx)	No complications
Sindel et al. 2018 [27]	-	–	–	1.77 mm	90%	–	1 IOI and BR failure
Stevenes et al. 2010 [28]	–	–	–	–	100%	–	No complications
Tekin et al. 2011 [29]	–	–	–	–	100%	Radiological stability between baseline and 6 years (periapical Rx)	No complications
Wychowansky et al. 2020 [11]	–	*a) BR group:* 4.3 ± 1.3 mm *b) XG group:* 4.4 ± 1.5 mm	–	–	*a) BR group:* 86,66% *b) XG group:* 96,66%	Implant stability at baseline (periotest) *a) BR group: −* 3.2 ± 1.3 *b) XG group: −* 1.2 ± 1.6 *(p<0,005) Implant stability at 24 m (periotest) *a) BR group: −* 3.7 ± 1.1 *b) XG group: −* 3.6 ± 1.2 *(p < 0.005)	*a) BR group:* -4 IOI failure *b) XG group:* -1 IOI failure
Yuce et al. 2019 [12]	–	–	–	–	100%	–	1 BR failure (defect repaired and IOI osseointegrated)

*BR* bone ring, *SB* sticky bone, *MBL* marginal bone loss, *IOI* implant, *HU Hounsfield units*, *XG* xenograft

### Study characteristics (Table 1)

All the studies together included a total of 186 patients treated with 266 implants. Out of the total of 266 implants, 219 were placed with BRT and 47 with other vertical ridge augmentation procedures. Among the 219 implants placed with BRT, 121 cases corresponded to an autogenous bone ring, of which 118 were harvested from the chin and 3 from the ramus. In the other 98 cases, an allogeneic bone ring was used. All the studies performing vertical ridge augmentation with allogeneic bone ring used resorbable collagen membranes to cover the grafted area. In one study, a platelet-rich fibrin (PRF) membrane was placed to cover the area grafted with an autogenous bone ring [21]. Regarding the intervention, BRT was used for vertical ridge augmentation in 14 studies, while in 2 studies BRT was used for sinus floor elevation. In two studies, BRT was compared with other regeneration procedures [10, 11]. The studies had a mean follow-up period of 17.17 ± 11.65 months. The longest follow-up was 3.1 years [18] and the shortest was 6 months [10, 19, 22, 24, 28].

### Results of individual studies (Table 2)

#### Bone gain

Bone gain evaluated through cone beam computed tomography (CBCT) was registered in four of the studies [10, 11, 21, 24]. The maximum bone height gain reported was 13.07 mm [24] and the minimum was 3.09 mm [10] with an overall mean of 4.94 mm across the four studies. Regarding comparative studies, Chandra et al. [10] found significant differences in terms of bone gain for BRT compared with GBR with sticky bone technique (*p* < 0.001) (Table 1), while Wychowansky et al. [11] registered a higher bone gain for vertical tunnel bone augmentation with xenograft compared with BRT.

#### Bone resorption

Two studies assessed bone resorption after BRT. The maximum value recorded was 0.94 ± 0.86 mm [24] and the minimum 0.78 ± 0.23 mm [21] with a mean bone resorption 0.83 mm.

#### Marginal Bone Loss (MBL)

Mean MBL of 0.57 mm was obtained after a mean follow-up period of 13.35 months. The highest value was reported by Sindel et al. [27] with 1.77 mm MBL after 24 months, while the minimum was 0.43 after a 12-month follow-up reported by Nord et al. [23].

#### Bone ring survival rate

Six bone ring failures among the total of 219 bone rings placed were recorded, with a mean bone ring survival rate of 97.26%. All the failures registered affected autogenous bone rings. Consequently, the allogeneic bone ring survival rate was 100%, while the autogenous bone ring survival rate was 95.04%.

#### Implant survival rate

The implant survival rate ranged between 86.6% and 100% across the studies with a mean survival rate of 94.97% (208 out of 219). Regarding the intervention studied and the type of bone ring used, implants placed with autogenous bone rings for vertical ridge augmentation procedures showed a 92.51% survival rate after a mean follow-up of 17.01 months. Implants placed with autogenous bone rings for sinus floor elevation obtained a 92.85% survival rate after 27.64 months follow-up; and the highest survival rate was found with allogeneic bone rings, with 97.93% after a mean follow-up period of 12.70 months.

#### Complications

Among the 219 implants placed with BRT, 11 osseointegration failures were recorded (5.03%). In addition, the studies reported three cases of swelling (1.94%), two cases of transient numbness of the lower lip (1.29%), and one wound dehiscence (0.64%) among the patients treated with BRT.

### Risk of bias (Tables 3 and 4)

Based on the Newcastle-Ottawa scale [14] two studies showed good quality, six studies were classed as presenting fair quality while two studies [21, 24] were rated as poor quality mainly due to their short follow-up periods. All the case reports included in this review showed a low risk of bias according to the Joanna Briggs Institute Critical Appraisal tool [16].

**Table 3:** Quality assessment of observational studies using the Newcastle-Ottawa scale

	Wychowansky et al. 2020 [11]	Yuce et al. 2019 [12]	Nord et al. 2019 [23]	Giesenhagen et al. 2019 [20]	Chandra et al. 2019 [10]	Sindel et al. 2018 [27]	Giraddi et al. 2017 [21]	Rizzo et al. 2017 [26]	Flanagan et al. 2016 [17]	Omara et al. 2016 [24]	Fukuda et al. 2005 [18]
**Selection**											
• Representativeness of the exposed cohort	*	*	*	*	*	*	*	*	*	*	*
• Selection of the non-exposed cohort	*	*	*	*	*	*	*	*	*	*	*
• Ascertainment of exposure	*	*	*	*	*	*	*	*	*	*	*
• Demonstration that outcome of interest was not present at start of study	*	*	*	*	*	*	*	*	*	*	*
**Comparability**											
• Study controls for bone ring group	*				*						
• Study control for any additional factor (duration of exposure)	*				*						
**Outcome**											
• Assessment of outcome	*	*	*	*	*	*	*	*	*	*	*
• Was follow-up long enough for outcomes to occur?	*	*	*	*		*		*	*		*
• Adequacy of follow up of cohorts	*	*	*	*		*		*	*		*
**Newcastle-Ottawa scale**	**9**	**7**	**7**	**7**	**9**	**7**	**5**	**7**	**7**	**5**	**7**

**Table 4:** Quality assessment of case reports using the Joanna Briggs Institute Critical Appraisal tools

***Study***	Miller J. 2019 [22]	Giesenhagen et al. 2018 [19]	Tekin et al. 2011 [29]	Stevenes et al. 2010 [28]	Peñarrocha et al. 2005 [25]
**Were patient´s demographic characteristics clearly described?**	+	+	+	+	+
**Was the patient’s history clearly described and presented as a timeline?**	+	−	−	−	+
**Was the current clinical condition of the patient on presentation clearly described?**	+	+	+	+	+
**Were diagnostic tests or assessment methods and the results clearly described?**	−	+	?	+	+
**Was the intervention or treatment procedure clearly described?**	+	+	+	+	+
**Was the post-intervention clinical condition clearly described?**	+	+	+	+	+
**Were adverse events (harms) or unanticipated events identified and described?**	+	+	+	+	+
**Does the case report provide takeaway lessons?**	−	+	+	+	+

+ = yes, − = no, ? = unclear

## Discussion

This systematic review aimed to analyze the clinical efficacy of BRT for restoring vertical alveolar ridge defects with simultaneous dental implant placement in terms of bone gain, bone resorption, MBL, survival rates of bone rings and implants, and complications. To the best of the authors’ knowledge, this is the first systematic review to evaluate the clinical outcomes of BRT.

A total of 186 patients treated with 266 dental implants were included in the review. Of the total 266 implants, 219 were placed with BRT. In 121 cases an autogenous bone ring was used, while the other 98 cases were performed using an allogeneic bone ring.

Unlike most of the techniques described for vertical ridge augmentation, the main clinical advantage of BRT is the possibility of a simultaneous approach, reconstructing a vertical alveolar ridge defect at the same time as implant placement, which reduces treatment time considerably. In addition, placing allogeneic bone rings can reduce or eliminate several complications related to autogenous bone block harvesting, such as the higher morbidity, the need for a donor site, neurosensorial disturbances, and the limited amount of bone available, among others [30, 31].

The most common complications observed after BRT were swelling (1.94%), transient numbness of the lower lip (1.29%), and wound dehiscence (0.64%). In any case, according to these findings, BRT showed a low rate of complications regardless of the type of bone ring used.

Nevertheless, BRT presents certain drawbacks: firstly, a minimum of 3–4 mm of apical native bone are required to stabilize the implant and the bone ring [32]; and secondly, most of the cases published describe single implants, and so there is a lack of evidence for the efficacy of BRT in large defects and multiple implant placement.

Regarding bone gain and subsequent resorption, a mean gain of 4.94 mm and mean bone resorption of 0.83 mm were observed. Consequently, a vertical bone gain of around 4 mm can be expected with BRT. Similar results have been described in a recent systematic review which evaluated the effectiveness of vertical ridge augmentation using various different techniques, which obtained a mean vertical bone gain of 4.16 mm [7].

According to Urban et al. [7], mean MBL of 1.01 can be expected around implants placed in augmented sites during the first year. The studies included in the present review showed a mean MBL of 0.57 mm after a mean follow-up of 13.35 months. So, based on the present findings, BRT would appear to undergo less resorption than other vertical GBR procedures.

The mean survival rate obtained in the present review was 97.26%; with 100% for allogeneic bone rings and 95.04% for autogenous bone rings. These outcomes are similar to other vertical ridge augmentation procedures [7, 30, 33].

Of the 219 dental implants placed with BRT analyzed in the review, the survival rate of implants placed with BRT was 94.97% after a mean follow-up of 17.17 ± 11.65 months. It should be noted that the highest values were found with allogeneic bone rings, with a 97.93% survival rate after a 12.70-month follow-up. These findings agree with previous systematic reviews analyzing implant survival rates after GBR and bone block grafting, which report survival rates of between 97% and 100% [5–7, 30].

Regarding the surgical technique, the included studies presented a lack of homogeneity. Therefore, further investigation of many aspects of BRT are needed in order to establish a clear and validated protocol regarding the use of membranes or whether or not to perform the technique as a single stage or in a staged approach. In this sense, animal studies have demonstrated that the use of membranes in BRT does not appear to offer any clinical advantage [34, 35], and no clinical differences have been observed between simultaneous implant placement or a staged procedure [36, 37].

In spite of the promising results for survival rate, bone gain, MBL, and the rate of complications, no long-term RCTs have compared BRT with other vertical ridge augmentation procedures. Moreover, the studies included in this review presented a lack of homogeneity in the surgical protocols followed. Therefore, there is insufficient evidence to reach any firm conclusions about long-term predictability of BRT.

## Conclusions

According to the findings of this systematic review, it may be concluded that BRT could be a valid option for restoring single vertical defects with dental implants in terms of bone gain, implant survival rates, and complications. BRT with simultaneous to dental implant insertion aims to shorten treatment time and reduce morbidity, especially when an allogeneic bone ring is used. However, further studies comparing BRT with other vertical ridge augmentation procedures with longer follow-up periods are needed in order to value the efficacy of BRT in different clinical scenarios.

## Data Availability

All the data generated in the course of this systematic review were included in the manuscript.

## Abbreviations

GBR: Guided bone regeneration
BRT: Bone ring technique
PRF: Platelet-rich fibrin
CBCT: Cone beam computed tomography
MBL: Marginal bone loss
